# The Pathological Buying Screener: Development and Psychometric Properties of a New Screening Instrument for the Assessment of Pathological Buying Symptoms

**DOI:** 10.1371/journal.pone.0141094

**Published:** 2015-10-21

**Authors:** Astrid Müller, Patrick Trotzke, James E. Mitchell, Martina de Zwaan, Matthias Brand

**Affiliations:** 1 Department of Psychosomatic Medicine and Psychotherapy, Hannover Medical School, Hannover, Germany; 2 General Psychology: Cognition, University of Duisburg-Essen, Duisburg, Germany; 3 Department of Clinical Psychiatry and Behavioral Science, University of North Dakota, School of Medicine and Health Sciences, Fargo, United States of America; 4 Neuropsychiatric Research Institute, Fargo, United States of America; 5 Erwin L. Hahn Institute for Magnetic Resonance Imaging, Essen, Essen, Germany; University of Ariel, ISRAEL

## Abstract

The study was designed to develop a new screening instrument for pathological buying (PB), and to examine its psychometric properties in a large-scale sample. By using a facet theoretical approach and based on literature as well as on clinical experience, a 20-item Pathological Buying Screener (PBS) was developed and administered to a representative German sample (*n* = 2,539). Valid data were available from 2,403 participants who were subjects for three subsequent empirical studies. The first study explored the factor structure using exploratory factor analyses in a subsample of 498 participants. Based on factor loadings, a 13-item version with the two factors *loss of control / consequences* and *excessive buying behavior* was revealed. This two-factor model was confirmed in study 2 by confirmatory factor analysis performed on another subsample (*n* = 1,905). Study 3 investigated age and gender effects and convergent validity of the PBS using the Compulsive Buying Scale (CBS) in the full sample (*N* = 2,403). The total PBS score was adequately correlated with the CBS score. Hierarchical regression analyses with the CBS score as the dependent variable and the two PBS factors as the predictors indicated an own incremental validity of the two factors in participants ≤ 65 years. The reliability of the total score as well as of the two subscales was good to excellent. Overall, the PBS represents a useful measure for PB. Future studies are needed to replicate the two-factor structure in clinical samples and to define a valid cutoff for PB.

## Introduction

Impulsively driven *oniomania* that results in large debts was first described more than 100 years ago by the German psychiatrist Emil Kraepelin [1]. Current representative surveys revealed point prevalence estimates of maladaptive excessive buying between 5.8 and 7% [2–6]. However, the questions, as to whether or not pathological buying can be viewed as a clinical condition and how it should be categorized are still a matter of debate. The ongoing academic discourse is reflected by the variety of terms for the problem, e.g. addictive, pathological or compulsive buying, the last of which is most commonly used in the psychiatric literature. Considering the disagreement about the proper categorisation we prefer the broader term *pathological buying* (PB), which will be used hereinafter rather than compulsive buying.

PB refers to extreme preoccupation with shopping and buying resulting in repetitive purchasing of mainly unnecessary consumer goods that are then rarely or never used [7]. Maladaptive spending serves as a way to manage negative mood states, to escape from anxiety and tension and to enhance poor self-esteem [7–11]. In the long run, the inappropriate buying behavior leads to severe distress, interference with social or occupational functioning, financial difficulties including bankruptcy, and sometimes delinquency [12]. In accordance with the proposed diagnostic criteria of McElroy et al. [7], mania should be ruled out as a reason for the buying binges. Treatment seeking patients with PB suffer from high psychiatric comorbidity, especially anxiety and depressive disorders, hoarding disorder, binge eating disorder, substance use disorders, and personality disorders [12].

In the light of some overlap in phenomenology of PB with other behavioral addictions such as gambling disorder or Internet gaming disorder [13–15], the categorization of PB as a non-substance related addiction has been introduced by several authors, but is still considered controversial. Support for the categorization as a behavioral addiction is seen in characteristics of PB. Among these, cue reactivity as a correlate of PB tendencies, craving and extreme preoccupation with buying or shopping, repetitive loss of control, the use of buying to relieve negative mood states, deceiving others regarding the extent of the inappropriate spending behaviour, jeopardizing significant relationships due to the inappropriate spending, relying on others to provide money to relieve desperate financial situations caused by repetitive buying binges, persistence and recurrence of the inappropriate spending behavior despite adverse consequences, and repeated unsuccessful attempts to limit buying, are the core characteristics of the phenomenon described in previous studies [13,16,17]. Alternatively, some authors favor the categorization of PB as an impulse control disorder, particularly due to high impulsivity and recurrent failure to resist an impulse to perform an act (i.e. excessive spending) that is harmful to the person or to others [12].

Even though there is consensus that the diagnosis of PB at best requires direct clinical assessment [18,19], the advantages of questionnaires to screen for the problem are obvious. Individuals with PB are often secretive and ashamed about their inappropriate spending behavior, and usually do not talk openly about it but may be willing to answer corresponding items on a self-rating instrument. Moreover, questionnaires are time-saving, easy to use and enable researchers to conduct large-scale surveys.

Several self-rating instruments to measure PB symptoms already exist (for review see [18,19]. However, these scales have shortcomings such as problems with the content of some items, constricting theoretical focuses, exclusion of items assessing psychological strain and other adverse consequences of PB. This might be caused by the fact that the questionnaires were developed mainly by consumer researchers and not by psychiatry researchers, and that most were developed in the late 1980s or early 1990s. Below, we briefly describe the most widely used questionnaires with empirically proven good psychometric properties.

According to the literature, the vast majority of studies [2,4] used the Compulsive Buying Scale (CBS) [20] to screen for PB. Faber and O’Guinn [19] developed this 7-item instrument based on preliminary studies using qualitative data (i.e., in-depth interviews with individuals with self-identified PB) and quantitative survey methods. The items are answered on a 5-point Likert scale (level of agreement or frequency). To test psychometric properties of the questionnaire, the authors collected data from 388 individuals with self-identified PB and from a general sample of 292 consumers. The CBS was posited as a unidimensional scale that assesses lack of impulse control, distress at the thought of others’ knowledge of the person’s purchasing habits, tension when not shopping, spending to feel better, and irrational use of credit cards or checks. A final score (i.e., beta weights from logistic regression for each item) can be calculated based on an algorithm, whereas a cutoff point at 2 SDs above the mean value in a general US population sample (*n* = 292) was used to diagnose PB. However, the dichotomous categorization of individuals with PB based on an arbitrary cutoff was repeatedly questioned [21]. Shortcomings of the CBS include concerns associated with the content of some items [21]. In the era of online banking and credit card use, the writing check item (item 2c: “Wrote a check when I knew I didn’t have enough money in the bank to cover”) does not match the current situation of many consumers, particularly of those outside the Unites States. Also, items 1a (“If I have any money left at the end of the pay period, I just have to spend it”) and 2f (“Made only the minimum payments on my credit card”) may be biased by age or culture. Other criticism concerned the proposed unidimensionality of the CBS. For example, Cole and Sherrell [22] examined the psychometric properties of the CBS in a convenience sample of 319 college students. The results indicated low factor loadings of the writing check item (0.44) and of item 2e (“I felt anxious and nervous on days I didn’t go shopping”, 0.37) when a single-factor confirmatory factor model was applied. Also, the average variance extracted across the seven items was low with 0.33 [22].

In response to the limitations of the CBS, Edwards [23] developed a scale addressing the dichotomous categorization of individuals with PB and the inclusion of items pertaining to credit card use and psychological aspects such as self-esteem. From her point of view, psychological aspects could serve to confound the assessment process. She developed an initial 29-item scale that was tested by using data from individuals with self-identified PB (*n* = 104) and from a convenience sample (*n* = 101) resulting in a final 13-item version. The five-point Likert scaled questions indicate frequency of behavior or level of agreement assessing a general tendency to spend, feelings experienced about and while shopping, and impulsiveness in purchasing, but not financial or money management aspects. Edwards’ scale measures the level of PB tendencies on the following five dimensions that are specifically linked to the dysfunctional behaviors surrounding spending behavior: *tendency to spend* (5 items), *compulsion/drive to spend* (2 items), *feelings about shopping and spending* (2 items), *dysfunctional spending* (2 items), and *post-purchase guilt* (2 items). Edwards concluded that these dimensions enable to characterize PB in a more fine-grained manner than the CBS. To our knowledge, the 13-item scale has not been utilized as extensively as the CBS and the statistical determination of subscale thresholds of the original version is pending. Maraz et al. [3] recently provided a cutoff score that, however, is based on a revised Hungarian version that consists of 16 (of the initial 29) items.

Ridgway, Kukar-Kinney and Monroe [24] published the Richmond Compulsive Buying Scale (RCBS) drawing on the theoretical concept of obsessive-compulsive spectrum disorders. The factor structure of the scale was initially examined in 352 undergraduate students and the questionnaire was then subsequently validated in 551 university staff members and 309 customers of an Internet women’s retailing store. Ridgway et al. aimed to combine the proposed impulse control (3 items, e.g., “I buy things I did not plan to buy”) and obsessive-compulsive (3 items, e.g., “Much of my life centers around buying things”) aspects of PB. The items are answered on a 7-point Likert scale (level of agreement or frequency). Those who on average score higher than the midpoint of all six items are categorized as compulsive buyers [24]. Similar to Edwards’ CBS, this questionnaire does not include items to assess adverse long-term negative consequences of inappropriate buying sprees. Ridgway et al. [24] argued that not all individuals with PB suffer from financial or psychiatric problems, and that those with PB tendencies but without negative long-term consequences have been neglected by other instruments. In our opinion, this approach is problematic given that the proposed diagnostic criteria we favor for PB clearly include the serious destructive effects on a person’s life (e.g., psychological distress, impairments, financial problems) [7,12,25]. While Ridgway et al. recommended using the scale in the general population and not solely in patients with PB, this instrument probably carries a risk of overestimating tendencies toward PB.

Most European studies [5,6,26] used the one-dimensional German Addictive Buying Scale (GABS) [27,28]. The GABS was modeled on the 13-item Canadian Compulsive Buying Measurement Scale [29], resulting in a final version with 16 items that are answered on a 4-point Likert scale (level of agreement). According to Raab et al. [28], the questionnaire allows one to distinguish between *compensatory* buying and *addictive* buying on the basis of cutoff scores of 1 or 2 SDs beyond the mean value of a German population-based sample. The questionnaire has been validated in representative German samples (e.g., in 1991: *N* = 1,527; in 2001: *N* = 1,017) [28]. It is noteworthy that the GABS is based on the concept that PB represents a behavioral addiction. Accordingly, the scale contains items pertaining to craving and to inner urgency to buy (e.g.,”I feel a strong urge to buy something”). Additionally, it contains items targeting post-purchase guilt, hiding of purchased goods or purchase of goods one cannot afford. The questionnaire is limited by the lack of questions about the resistance against PB behavior or about the degree of suffering from PB that are of relevance for diagnosing PB and for the use in treatment settings.

Taking into account the comorbidity of PB and compulsive hoarding, Frost et al. [30] created the Compulsive Acquisition Scale (CAS) which measures the extent to which individuals acquire and feel compelled to acquire possessions. The scale includes 12 questions with regard to PB (CAS-Buy: e.g., “Do you buy things you never use?”, “Do you buy things to make yourself feel better?”) and 6 questions with respect to the acquisition of free things (CAS-Free: e.g., “Do you pick things up that other people have discarded?”, “Do you look through other people’s trash (for example, dumpsters) for things to bring home?”). All questions are answered on a 7-point Likert scale indicating either level of agreement or frequency. However, the suggested 2-factor structure (CAS-Buy and a CAS-Free) of the German version could not be replicated in a population-based sample (*N* = 2,373) [31].

Given the limitations of existing questionnaires, the aims of the present study were: 1) to develop a new screening instrument incorporating characteristics of both behavioral addictions and impulse control disorders; and 2) to investigate psychometric properties -particularly dimensionality, reliability, and congruent validity—of the new questionnaire in a large-scale German population-based sample.

## Development of the Pathological Buying Screener (PBS)

We employed a facet theoretical approach to develop the questionnaire [32]. Based on literature reviews including empirical studies with both patients with PB and non-clinical samples as well as diagnostic criteria of both behavioral addictions and impulse control disorders, the following aspects of PB were defined: preoccupation / craving, loss of control, emotion regulation, not using purchased goods / hiding purchases / lying about spending / deception, degree of suffering, interference with other life aspects and financial aspects / consequences, and resistance against excessive spending. Afterwards, items were created with respect to these aspects, based on clinical experience with PB over more than 20 years (i.e. clinical interviews, psychotherapy, discussion with assessors, therapists and patients’ relatives) and the relevant literature.

Initially, 33 items were selected as potential candidates for use. All items asked respondents to indicate the frequency (“How often does it occur…?”) of experiencing feelings, thoughts, behaviors or consequences of buying on a 5-point Likert scale ranging from 1 (“never”) to 5 (“very frequently”). In order to make a reasonably precise diagnosis all items referred to the specific time period of past six months. This time frame was determined with the episodic course of the disorder in mind. After careful pretests of the item wording by evaluations from experts in the field, item characteristics and factor structure of the initial 33-item version of the PBS was tested in a predefined sample of 119 participants (mainly students) and a sample of 19 patients suffering from PB. Of the 119 non-clinical participants, 76 were female and 46 male. The mean age was 26.7 years (*SD* = 7.6, *range* 19 to 59) and the average school education ranged from 8 to 13 years (m*edian* = 13). The patients sample comprised of 16 female and 3 male participants with a mean age of 45.5 years (*SD* = 10.7, *range* 20 to 60) and an education ranging from 9 to 13 years (*median* = 10). Based on these preliminary data, we adapted the instruction and made some revisions in the item pool considering item characteristics (i.e., double-barreled or ambiguous items) and exploratory factor analysis. The resulting version of the PBS consisted of 20 items.

In addition to these 20 items, we created three supplementary items that could be useful for additional assessments. The first item asks about possible symptoms of mania (M) that should be ruled out as a reason for buying episodes [7]. Since PB is often accompanied by compulsive hoarding that might negatively influence the course of the disorder [30,33], the second supplementary item refers to hoarding (H). The third supplementary item was included to give a first hint at excessive buying with the primary goal of personal enrichment (E) that should be delineated from PB. The three items were “How often does it occur … (M) that you are in a high mood, and that you get into difficulties when this happens? / (H) that you cannot get rid of things, so that clutter develops? / (E) that you buy something in order to resell it for a profit?”.

The final instrument consisting of the 20 PB items and the three additional MHE-items was subject for the empirical studies described below. In order to provide not only a German but also an English version of the instrument, all items were translated into American English by a licensed translator (Translaw, Oxford, United Kingdom). The English version was then verified by one of the coauthors (J.E.M.) who is a native speaker and a researcher on PB.

## Procedure

### Ethics Statement

The survey met the ethical guidelines of the International Code of Marketing and Social Research Practice by the International Chamber of Commerce and the European Society for Opinion and Marketing Research. The study was approved by the ethics committee of the University of Leipzig.

### Data Sampling

Data were collected between February and April 2014. A random sample of the German general population older than 14 years of age was selected with the assistance of a demographic consulting company (USUMA, Berlin, Germany). The sampling procedure followed the established guidelines on how to construct a random population sample in Germany when no access to a population roster is possible. This sampling design involves three consecutive steps: in the first step, a grid of 258 regional sampling areas was randomly selected from a roster of such non-overlapping grids that have been centrally assembled to enhance representativeness in stratified regional sampling in Germany. In the second step, a random procedure to select households of the respective area was implemented within all sampling areas. In the final step, one member of the selected household fulfilling the inclusion criteria (age 14 or older, able to read and understand the German language) was sampled randomly in a pre-specified standardized manner. The sampling procedure is designed to yield random samples representative in terms of age, gender, and education of the German population. A first attempt was made for 4,644 addresses, of which 4,607 were valid. If not at home, a maximum of three attempts was made to contact the selected person. All subjects were visited by a study assistant who informed them about the investigation, obtained written informed consent, and presented them with the questionnaire.

A total sample of 2,539 individuals (55.1% of valid addresses) agreed to participate. Cases with missing values within the applied questionnaires were removed resulting in a final sample of 2,403 participants without any missing data. Table 1 displays sociodemographic characteristics of the sample. For subsequent analyses the sample was divided into two subsamples by applying stratified probability sampling, at random, with respect to age and gender. This procedure ensured that the representative characteristics of the population based survey approximately remained within each subsample. Randomized division of this large sample into two subsamples had the advantage that different samples were used for conducting exploratory factor analysis (EFA) and confirmatory factor analysis (CFA). Convergent validity and preliminary cutoff scores were investigated using the total sample (*N* = 2,403).

**Table 1:** Sociodemographic characteristics of the total sample (*N* = 2,403).

	mean *(SD)*
Age [years]	49.2 (17.7)
	*n* (%)
Age Groups [years]	
≤ 24	251 (10.4)
25 to 34	325 (13.5)
35 to 44	380 (15.8)
45 to 54	465 (19.4)
55 to 64	450 (18.7)
65 to 74	342 (14.2)
≥ 75	190 (7.9)
Gender	
male	1121 (46.7)
female	1282 (53.3)
Marital status	
married	1157 (48.1)
single	661 (27.5)
divorced	336 (14.0)
widowed	247 (10.3)
n/a	2 (0.1)
Nationality	
German	2314 (96.3)
Other	89 (3.7)
School years	
≥ 12 years	470 (19.6)
< 12 years	1932 (80.4)
Monthly household income [Euro]	
< 1250	439 (18.3)
1250 to < 2500	1022 (42.5)
≥ 2500	879 (36.6)
n/a	63 (2.6)

### Instruments

The 20-item version of the PBS with a total score range from 20 to 100 and with three supplementary diagnostic items was administered to all subjects and the relative order of items was the same in studies 1, 2 and 3.

To explore congruent validity (study 3), we administered the German version [4] of the CBS [20]. In accordance with Faber and O’Guinn [20], the items were weighted to obtain the total score. Lower scores indicate a higher level of PB symptoms. According to a prior representative German survey [4], a cutoff score equal to -1.09 or lower defines individuals as being at-risk for PB. Cronbach’s *α* of the CBS in the total sample was 0.886.

## Study 1

The aim of the first study was to extract the number of factors of the PBS using EFA and–if necessary–to modify the scale with respect to the number of items and further methodological issues.

### Methods

#### Participants

The first study included 498 participants of the total sample by using stratified probability sampling, at random. Of the 498 participants, 231 were male and 267 were female. The average age was 49.0 years (*SD* = 17.4, *range* 14 to 88) years, and school education ranged from 8 to 13 years (*median* = 10).

#### Statistical analyses

Exploratory factor analyses were conducted by SPSS version 22.0 for Windows (IBM SPSS Statistics). Horn's parallel analysis [34] was used to determine the appropriate number of factors. As suggested by Zwick and Velicer [35], we also added a randomly generated unique variable to the data set to additionally ensure the appropriateness of the number of factors.

#### Results

An EFA with principal axis analysis and promax rotation was conducted to assess the dimensionality of the PBS and to determine the number of factors extracted. In a second step, an EFA with principal axis analysis and varimax rotation and the numbers of factors obtained by parallel analysis was conducted to prove the factor loadings.

**Fig 1:**
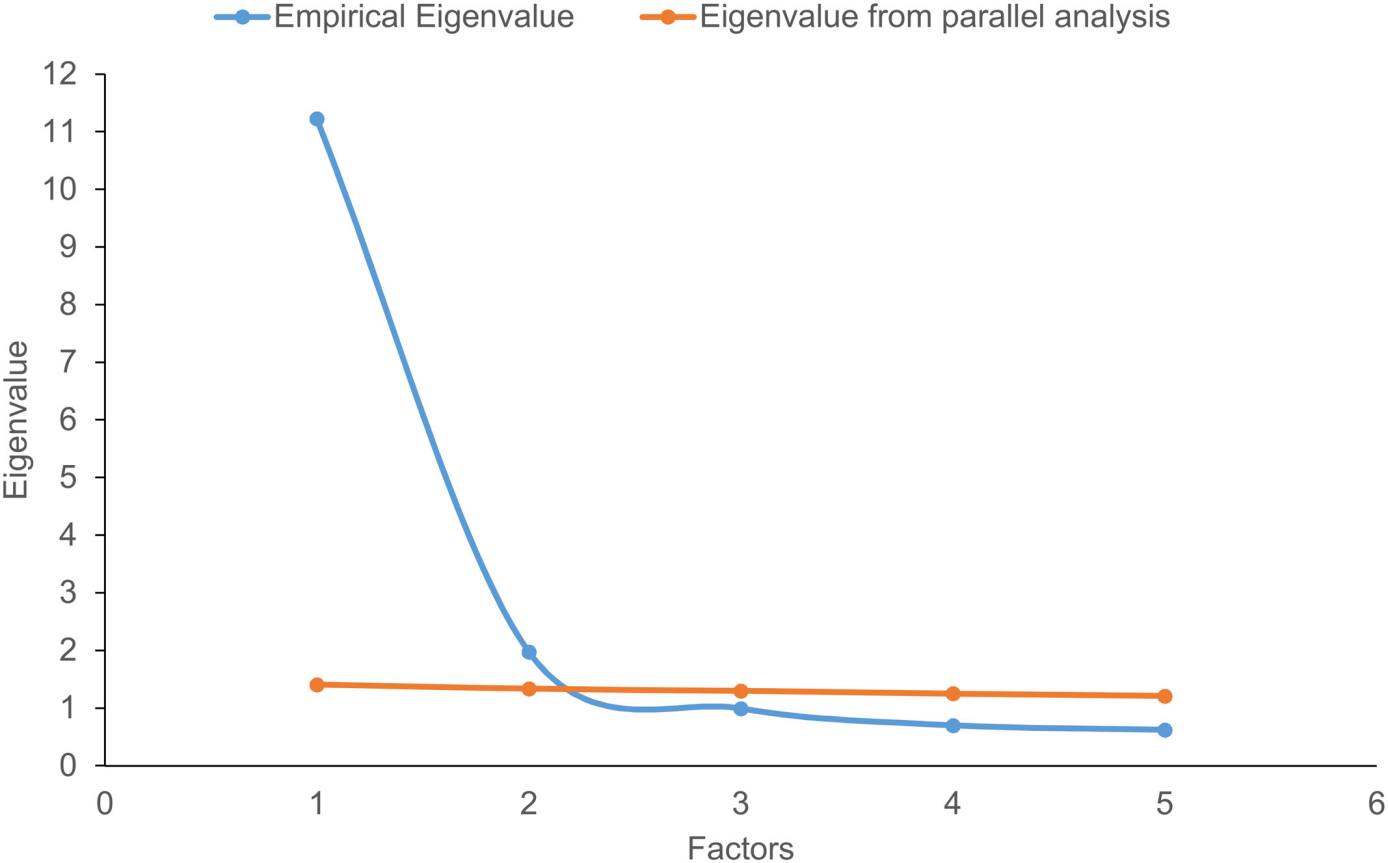
Empirical eigenvalues of the first and the second factor.

The criteria of Horn's parallel analysis [34] suggested a two-factor solution. This was also confirmed by the method suggested by Zwick and Velicer [35] in a second analysis including a random variable (normally distributed with *mean* = 3 and *SD* = 1), given that the randomly generated variable added to the data set did not load on any of the factors extracted (loading of 0.019 on the first and -0.072 on the second factor). Thus, a two-factor solution was appropriate and consequently only eigenvalues exceeding the eigenvalue derived in the parallel analysis were extracted. Also, even when applying the Kaiser criterion (eigenvalue higher then 1) a two-factor solution was the result, because only two eigenvalues reached this criterion. The empirical eigenvalues of the first and the second factor were higher compared to the eigenvalue obtained in the parallel analysis. The third factor was not extracted, because the empirical eigenvalue was lower than 1, and it was not higher than the eigenvalue obtained in the parallel analysis (see Fig 1). The two-factor solution explained 66.02% of the variance.

The EFA with principal axis analysis and varimax rotation was conducted to prove the factor loadings for each item in the two-factor solution of the PBS. Items with suboptimal loadings were excluded to enhance the validity and reliability of the PBS on the basis of empirical based criteria [36]. Items with low main loadings (< 0.50) and / or high parallel loadings (> 0.20) were excluded from further analysis [37]. In accordance with this procedure, we have excluded 7 items, because of either low main loadings and / or high parallel loadings (for factor loading of all 20 items, see S1 Table). From a conceptual point of view, none of the deleted items made uniquely crucial, non-redundant contribution to the instrument.

After excluding the 7 items and again conducting the EFA, the final 13-item version of the PBS with a two-factor structure remained (see Table 2). The first factor contains 10 items with high loadings on the main factor (> 0.50) and low loadings on the other factor (< 0.20) and relates to subjective complaints linked to buying behavior such as preoccupation, loss of control over buying, social and financial problems and mood regulation, so we named this factor *loss of control / consequences*. The second factor consists of three items with high loadings on the main factor (> 0.50) and low loadings on the other factor (< 0.20), and all items relate to excessive buying behavior, so we called this factor *excessive buying behavior*.

**Table 2:** Factor loadings and means of the rated items for the 13-item Pathological Buying Screener, Study 1 (*n* = 498).

Item No.	Item: English translation (German version)	Factor 1	Factor 2	*Mean*	*SD*
	How often does it occur… (Wie oft kommt es vor, …)				
	***Factor 1*: *Loss of Control / Consequences***				
Q 6	…that you have problems at work or school or in other areas due to your buying behavior?				
	(dass Sie aufgrund Ihres Kaufverhaltens Probleme in beruflichen, schulischen oder anderen Lebensbereichen haben)	**0.924**	-0.083	1.14	0.512
Q 12	…that you try to limit your buying and can’t?				
	(dass Sie versucht haben, ihr Kaufverhalten einzuschränken und es nicht schaffen)	**0.881**	0.003	1.16	0.547
Q 9	…that you hide your buying habits from others?				
	(dass Sie Ihr Kaufverhalten vor anderen verbergen)	**0.877**	-0.057	1.16	0.544
Q 11	…that you cannot stop buying things despite financial problems?				
	(dass Sie trotz finanzieller Probleme nicht aufhören können zu kaufen)	**0.866**	-0.030	1.16	0.532
Q 13	…that you have problems with other people due to your buying habits?				
	(dass Sie mit anderen Menschen aufgrund Ihrer Kaufgewohnheiten Probleme haben)	**0.861**	-0.07	1.15	0.551
Q 5	…that you suffer distress from your buying habits?				
	(dass Sie unter Ihren Kaufgewohnheiten leiden)	**0.842**	-0.018	1.16	0.533
Q 3	…that you have financial difficulties due to your buying habits?				
	(dass Sie durch Ihr Kaufverhalten unter finanziellen Problemen leiden)	**0.793**	0.043	1.25	0.632
Q 2	…that you feel embarrassed when others ask you about your buying behavior?				
	(dass es Ihnen unangenehm ist, wenn andere Sie auf Ihr Kaufverhalten ansprechen)	**0.729**	0.056	1.22	0.618
Q 8	…that at times you don’t feel good and that you feel better when you go buying?				
	(dass es Ihnen schlecht geht und sich das bessert, wenn Sie einkaufen)	**0.694**	0.164	1.30	0.706
Q 1	…that you can’t stop thinking about buying?				
	(dass Sie ständig ans Kaufen denken müssen)	**0.672**	0.149	1.17	0.508
	***Factor 2*: *Excessive Buying Behavior***				
Q 10	…that you buy more than you had planned?				
	(dass Sie mehr kaufen, als Sie sich vorgenommen haben)	-0.038	**0.851**	1.87	0.907
Q 7	…that you buy more things than you need?				
	(dass Sie mehr Dinge kaufen als Sie benötigen)	0.048	**0.847**	1.80	0.919
Q 4	…that you spend more time buying than you intended?				
	(dass Sie länger einkaufen als beabsichtigt)	-0.010	**0.763**	1.77	0.984

*Note*. The translation of the German version into American English was performed by a licensed translator.

In order to detect redundant items with extremely high inter-correlations, we analyzed the inter-item-correlation-matrix and revealed quite appropriate correlations, ranging from *r* = 0.245 to *r* = 0.694. Items Q6 (‘problems at work or school’) and Q13 (‘problems with other people’) were rather highly correlated with *r* = 0.757. From a clinical point of view, we assumed that both items measure only partly overlapping consequences and that one item may provide information beyond the other one. Consequently, we did not remove further items.

Both factors had good reliability (Cronbach’s *α* = 0.951 for *loss of control / consequences* and *α* = 0.857 for *excessive buying behavior*). The reliability for the PBS overall score was also very good (Cronbach’s *α* = 0.923).

## Study 2

The purpose of study 2 was to confirm the two-factor structure of the 13-item version of the PBS using a CFA performed on another sample than the EFA.

### Methods

#### Participants

For the CFA, we included the remaining 1,905 participants of the total sample (890 males and 1,015 females). These participants were not included in the sample of study 1. The average age was 49.3 years (*SD* = 17.8, *range* 14 to 95), and school education ranged from 8 to 13 years (*median* = 10).

#### Statistical analyses

The CFA was done with MPlus [38]. We applied the following standard criteria for the evaluation of well-established model fits [39,40]: the standardized root mean square residual (SRMR; values below 0.08 indicate good fit with the data), comparative fit indices (CFI/TLI; values above 0.90 indicate a good fit, values above 0.95 an excellent fit), and root mean square error of approximation (RMSEA; “test of close fit”; a value below 0.08 with a significance value below 0.05 indicates acceptable fit). Although some additional model fit criteria have been suggested [41], we started with using the standard criteria and checked whether certain corrections for large samples were necessary (which was not the case). Cronbach’s *α* and discriminatory power of the items were calculated with SPSS version 22.0 for Windows (IBM SPSS Statistics).

#### Results

**Fig 2:**
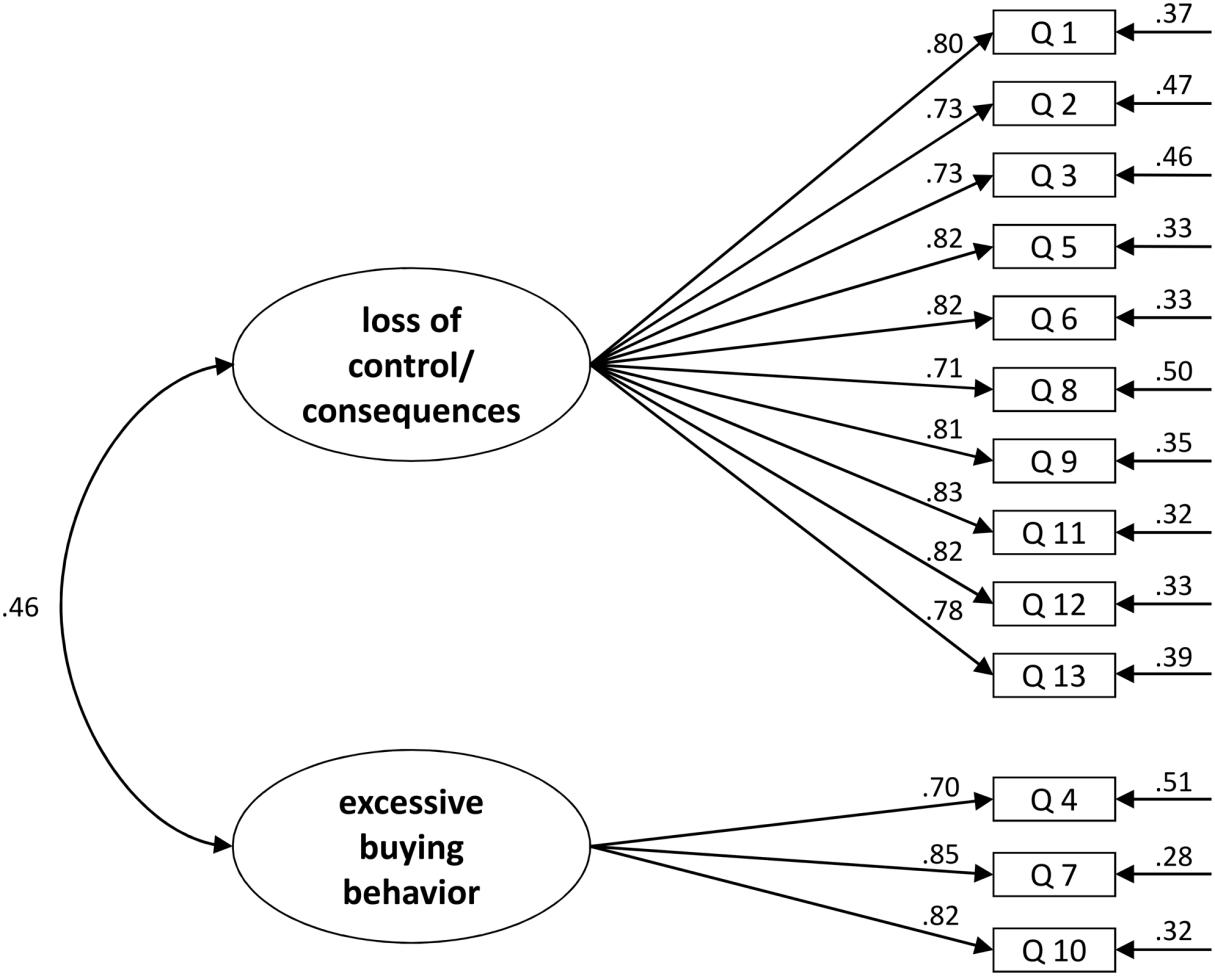
Results of the confirmatory factor analysis, study 2 (*n* = 1,905). RMSEA = 0.08, CFI = 0.96, TLI = 0.95, and SRMR = 0.04.

The CFA confirmed the two-factor solution for the PBS (RMSEA = 0.08, CFI = 0.96, TLI = 0.95, and SRMR = 0.04) The RMSEA was a bit high and the *χ* ^2^-test was significant, *χ* ^2^(64) = 756.45, *p* < 0.001. The completely standardized loadings and the standardized residuals are shown in Fig 2.

The two factors of the PBS were significantly correlated (*r* = 0.429, *p* < 0.001). Both factors had high internal consistency: *loss of control / consequences α* = 0.936, *excessive buying behavior α* = 0.832. Cronbach’s *α* for the PBS total score was 0.901. When considering the two factors separately, all items had very high discriminatory power (0.68 to 0.80 for the items of the factor *loss of control / consequences* and 0.63 to 0.73 for *excessive buying behavior*). When calculating the discriminatory power of the items for the whole scale, values were still good to very good (0.49 to 0.73). The average extracted variance was 0.62 for the factor *loss of control / consequences* and 0.63 for the factor *excessive buying behavior* and therefore higher than the cutoff (> 0.50) suggested by Fornell and Larcker [42].

We additionally tested a one-factor solution, but the model did not show acceptable fit indices: RMSEA = 0.14, CFI = 0.85, TLI = 0.82, and SRMR = 0.09. The *χ* ^2^-test was significant, *χ* ^2^(65) = 2543.61, *p* < 0.001. Therefore, we kept the two-factor solution. However, as the two factors were correlated moderately, an overall sum score for the PBS could be calculated.

## Study 3

The purpose of study 3 was to evaluate convergent validity of the PBS using the CBS in the total sample (*N* = 2,403). In addition, this study aimed at defining preliminary cutoff scores for the PBS and at exploring the response frequencies of the supplementary MHE-items.

### Methods

#### Participants

Study 3 included the total sample (*N* = 2,403) which was already described in detail above (see Table 1).

#### Statistical analyses

Pearson correlations, hierarchical regressions and descriptive statistics were performed by SPSS version 22.0 for Windows (IBM SPSS Statistics).

### Results

#### Congruent validity

The mean PBS total score was 17.27 (*SD* = 5.74, *range* 13 to 57, *maximal range* 13 to 65) and the mean CBS score was 2.62 (*SD* = 1.72, *range* -7.03 to 3.61, *maximal range* -7.03 to 3.61). The overall PBS score was adequately correlated with the CBS score (*r* = -0.572, *p* < 0.001, shared variance 32.7%). Note that lower scores in the CBS indicate stronger PB symptoms, meaning that the inverse correlation represent the theoretically assumed relationship between the two scales. Both PBS subscales were moderately intercorrelated (*r* = 0.436, *p* < 0.001) and both PBS subscales were also correlated significantly with the CBS score. Interestingly, the correlation between the PBS factor *loss of control / consequences* and the CBS (*r* = -0.605, *p* < 0.001) was higher than the correlation between the PBS factor *excessive buying behavior* and CBS (*r* = -0.291, *p* < 0.001), although both correlations were significantly different from zero on a simple bivariate level. The difference between the two correlations was significant (*Fisher’s z* = 13.9, *p* < 0.001). The PBS factor *loss of control / consequences* and the CBS shared 36.6% of variance, while the PBS factor *excessive buying behavior* and the CBS shared significantly lower proportions of variance (8.5%).

We also analyzed the effects of age and gender for the CBS and the PBS in this large, representative sample. On a simple level, age was correlated with the CBS score (*r* = 0.123, *p* < 0.001) as well as with the PBS score (*r* = -0.142, *p* < 0.001) and both subscales of the PBS (*loss of control / consequences*: *r* = -0.108, *p* < 0.001; *excessive buying behavior*: *r* = -0.146, *p* < 0.001). Gender did not have an effect on the CBS score (women: *mean* = 2.58, *SD* = 1.66; men: *mean* = 2.65, *SD* = 1.79, *t* = -1.00, *p* = 0.315), but on the PBS total score (women: *mean* = 17.94, *SD* = 6.01; men: *mean* = 16.51, *SD* = 5.32, *t* = 6.18, *p* < 0.001). The gender effect was also found when inspecting the two subscales of the PBS separately: *loss of control / consequences* (women: *mean* = 12.00, *SD* = 4.47; men: *mean* = 11.45, *SD* = 3.98, *t* = 3.2, *p* = 0.001) and *excessive buying behavior* (women: *mean* = 5.94, *SD* = 2.5; men: *mean* = 5.06, *SD* = 2.24, *t* = 9.06, *p* < 0.001). While the significant gender differences had only mild effect sizes for both the total score of the PBS (*d* = 0.25) and the subscale *loss of control / consequences* (*d* = 0.13), the effect for the PBS factor *excessive buying behavior* was almost moderate (*d* = 0.38). We further calculated moderated regression analyses with gender, age, and the interaction of gender and age as predictors and the CBS, PBS, and the two subscales of the PBS as separate dependent variables. In none of these moderated regression analyses a significant interaction effect of gender and age was observed.

To ensure that both factors significantly contribute to common variance with the CBS, when corrected for inter-correlations between the two PBS factors, we conducted a hierarchical regression analysis. The CBS score was the dependent variable and the two PBS factors were the predictors in two steps. In the first step, the factor *loss of control / consequences* was a significant predictor of the CBS score, *R* ^*2*^ = 0.367, *F* (1, 2401) = 1389.55, *p* < 0.001. When adding (second step) the factor *excessive buying behavior* as a predictor, the changes in *R* ^2^ failed to reach significance, *ΔR* ^*2*^ = 0.001, *F* (1, 2400) = 3.47, *p* = 0.063. The overall model was significant, *F* (2, 2400) = 697.22, *p* < 0.001. The two PBS factors had the following statistic values: factor *loss of control / consequences β* = -0.591, *t* = -32.76, *p* < 0.001, partial correlation *r* = -0.556, factor *excessive buying behavior β* = -0.034, *t* = -1.86, *p* = 0.063, partial correlation *r* = -0.038. The results suggest that the shared variance with the CBS only relies on the PBS factor *loss of control / consequences* while the factor *excessive buying behavior* narrowly failed to explain the shared variance significantly (*p* = 0.06).

Considering the aforementioned age effect on both the CBS and the PBS, we divided the total sample into two age groups. The older age group included all participants of the total sample who aged above 65 years (*n* = 484) as this equals retirement ages in most developed countries. Accordingly, the younger age group consisted of participants who aged ≤ 65 years (*n* = 1,919). We then analyzed the increment of both PBS factors on the shared variance with the CBS by these two age groups. Again, the CBS score was the dependent variable and the two PBS factors were the predictors in two steps. For the age group > 65 years, we revealed the same results as in the total sample with *loss of control / consequences* being a significant predictor of the CBS score (first step) *R* ^*2*^ = 0.111, *F* (1, 482) = 60.14, *p* < 0.001. When adding (second step) the factor *excessive buying behavior* as a predictor, the changes in *R* ^2^ were again not significant, *ΔR* ^*2*^ = 0.001, *F* (1, 481) = 0.464, *p* = 0.496 with the significant overall model, *F* (2, 481) = 30.27, *p* < 0.001. The PBS factors had the following coefficients: factor *loss of control / consequences β* = -0.342, *t* = -7.6, *p* < 0.001, partial correlation *r* = -0.327, factor *excessive buying behavior β* = 0.031, *t* = 0.68, *p* = 0.496, partial correlation *r* = -0.031. Interestingly, in the younger age group (≤ 65 years) the second factor *excessive buying behavior* of the PBS added significant variance explanation within the model. The factor *loss of control / consequences* in the first step was again a significant predictor of the CBS score *R* ^*2*^ = 0.406, *F* (1, 1917) = 1309.15, *p* < 0.001. When adding the factor *excessive buying behavior* as a predictor in the second step, the changes in *R* ^2^ were significant, *ΔR* ^*2*^ = 0.001, *F* (1, 1916) = 4.49, *p* = 0.034. The effect was minimal, resulting in an overall common variance of both PBS factors and the CBS of 40.7%, *F* (2, 1916) = 658.02, *p* < 0.001. The PBS factors had the following coefficients: factor *loss of control / consequences β* = -0.618, *t* = -31.42, *p* < 0.001, partial correlation *r* = -0.583, factor *excessive buying behavior β* = -0.042, *t* = -2.12, *p* = 0.034, partial correlation *r* = -0.048.

#### Preliminary cutoff scores

We calculated preliminary cutoff scores in the total sample (*N* = 2,403). When using 2 SDs for defining a cutoff score for PB behavior, this score would be ≥ 29 indicating a point prevalence estimate of PB in our total sample of 4.8%. Of note, by using the German CBS cutoff for PB [4], 4.7% of the sample would be defined as suffering from PB, whereas the overlap between the PBS- and the CBS-based diagnoses is only moderate with *κ* = 0.494 (*p* < 0.001). In summary, 116 subjects were categorized as having PB by the PBS and 112 participants reached the cutoff of the CBS. However, only 59 subjects were diagnosed as having PB by both PBS and CBS, while 57 reached the cutoff of the PBS but not of the CBS and 53 participants reached the cutoff score of the CBS but not of the PBS.

An alternative method for defining a cutoff value is the assumption that all items measuring the frequency of problematic behavior or symptoms specified in a questionnaire should be rated on average as “sometimes” in all items [43]. The method was also used by Ridgway et al. [24] when defining a cutoff point for the RCBS. This approach would result in a PBS cutoff score for PB of 39 (i.e. 13 items and a rating scale from 1–5 with “3” representing “sometimes”). According to this cutoff point, the prevalence of PB would be estimated to be only 1.8%.

#### Response frequencies of the supplementary MHE-items

Item M (“How often does it occur that you are in a high mood, and that you get into difficulties when this happens?”) has been answered by 2,394 (99.6%) participants. The vast majority (87.6%) responded with “never”, 8.0% answered “rarely”‘, 3.4% “sometimes”, 0.5% “frequently”, and only 0.1% answered “very frequently”. Responses to item H (“How often does it occur that you cannot get rid of things, so that clutter develops?”) were available from 2,399 participants (99.8%). The responses were distributed as follows: 61.3% “never”, 22.2% “rarely”, 12.1% “sometimes”, 3.5% “frequently”, and 0.8% “very frequently”. With regard to item E (“How often does it occur that you buy something in order to resell it for a profit?”) which has been answered by 2,395 participants (99.7%), most frequent responses were “never” with 81.6%, followed by “rarely” (10.1%), “sometimes” (5.9%), “frequently” (1.6%), and “very frequently” (0.4%).

We also calculated the proportion of individuals with probable PB who answered the MHE-items with “frequently” or “very frequently”. Based on a preliminary PBS cutoff score of ≥ 29, the rates were 12.9% (15 of 116) for the M-item, 28.9% (33of 114) for the H-item, and 16.5% (19 of 115) for the E-item. The proportions were higher when applying a preliminary cutoff score of ≥ 39, in particular 30.2% (13 of 43) for the M-item, 38.1% (16 of 42) for the H-item, and 31.0% (13 of 42) for the E-item.

## Discussion

The present study aimed at developing a new screener to identify symptoms of PB and to investigate the dimensionality of the instrument in a large population-based sample. Below we discuss our findings with respect to psychometric properties, preliminary cutoff scores for PB, and the additional value of the new questionnaire.

### Psychometric properties of the PBS

The results of the EFA in a first subsample revealed a two-factor solution with the dimensions *loss of control / consequences* and *excessive buying behavior* (study 1). This model was confirmed by a CFA in another subsample (study 2). The congruent validity of the new instrument was investigated using the CBS in the full sample (study 3). Both questionnaires were adequately correlated. In all three studies, the reliability of the total score as well as of the two subscales was good to excellent. Hence, we conclude that the PBS represents a reliable and valid instrument that can be applied to assess PB symptoms.

### Considerations on preliminary cutoff scores

We applied various methods to suggest a threshold value for PB in the present sample. The first method (i.e., 2 SDs above the mean), which was also applied by the authors of the CBS [20] and the GABS [28], resulted in a cutoff point of ≥ 29 that lead to PBS prevalence estimates similar to those assessed with the CBS. However, the concordance between the PBS and the CBS based diagnoses was only moderate. This result could be explained by the fact that the CBS does not require a specific time frame for the diagnosis, while the PBS measures PB within the past six months which may have led to more precise prevalence estimates.

The second method (i.e. on average scoring on at least midpoint for all scale items), which was also favored for the RCBS [24], yielded a cutoff value of ≥ 39 resulting in a substantially lower point prevalence estimate of only 1.8%. Based on the present data we are not able to define which of the two cutoff points should be applied for clinical diagnosis. There is no doubt that the definition of a valid PBS cutoff score and its sensitivity and specificity requires further investigation by comparing PBS data of patients with diagnosed PB, other clinical samples, and healthy individuals.

### What is the additional value of the PBS?

The results of the hierarchical regression analyses in study 3 with the CBS score as the dependent variable and the two PBS factors as the predictors indicated an own incremental validity of the two PBS factors in adults ageing ≤ 65 years. This finding suggests that the combination of the two PBS factors goes beyond the CBS. Especially the second factor *excessive buying behavior* seems to provide some information that were not depicted by the CBS (indicated by moderate correlations with the CBS and age dependent explanation of the shared variance with the CBS).

Table 3 demonstrates overlaps and differences between the PBS, the CBS, and other—most widely used—screeners for PB. It is important to note that only the PBS and the GABS [28] have been validated in large-scale representative samples. As can be seen from the table, the new measure includes items referring to important aspects of PB that were not directly assessed by other questionnaires, namely to the interference from PB (i.e. problems at work or school or in other areas due to buying behavior), the resistance to PB (i.e. unsuccessful attempts to limit buying), and the degree of suffering from PB. The assessment of these aspects is of relevance when diagnosing the level of PB.

**Table 3:** Comparison of the Pathological Buying Screener and other questionnaires assessing pathological buying.

	Compulsive Buying Scale [20]	German Addictive Buying Scale [28]*	Richmond Compulsive Buying Scale [24]	Pathological Buying Screener
**Indicated time period**	n/a	n/a	n/a	past 6 months
**Proposed facets of pathological buying**				
Preoccupation / craving	n/a	Six items concerning the strong urge to buy something*	“I buy something for myself almost every day.”	Q 1 …that you can’t stop thinking about buying?
			“Much of my life centers around buying things.”	
Loss of control	n/a	n/a	“I buy things I did not plan to buy.”	Q 11…that you cannot stop buying things despite financial problems?
			“I buy things without thinking.”	Q 10…that you buy more than you had planned?
			“I am a bit reckless about what I buy.”	Q 4…that you spend more time buying than you intended?
Emotion regulation	“Bought something in order to make myself feel better.”	One item concerning escape from unpleasant everyday life*	n/a	Q 8…that at times you don’t feel good and that you feel better when you go buying?
	“Felt anxious or nervous on days I didn’t go shopping.”^1^			
Buying of unneeded goods / of more than needed / not using purchased goods	n/a	One item concerning buying something that remained unused*	“My closet has unopened shopping bags in it.”	Q 7 …that you buy more things than you need?
			“I buy things I don’t need.”	
Reaction of others / hiding purchases / lying about spending / deception	“Felt others would be horrified if they knew of my spending habits.”	One item concerning not daring to show purchased goods due to expected uncomfortable responses*	“Others might consider me a ‘shopaholic’.”	Q 9…that you hide your buying habits from others?
				Q 2…that you feel embarrassed when others ask you about your buying behavior?
Financial aspects	“If I have any money left at the end of the pay period, I just have to spend it.”	Three items concerning financial aspects*	n/a	Q 3…that you have financial difficulties due to your buying habits?
	“Bought things even though I couldn’t afford them.”			
	“Wrote a check when I knew I didn’t have enough money in the bank to cover it.”			
	“Made only minimum payments on my credit cards.”			
Post-purchase guilt	n/a	Two items concerning post-purchase doubts/guilt*	n/a	n/a
Self-concept about consume habits	n/a	One item concerning the self-perception of being wasteful * ^2^	“I consider myself an impulse purchaser.”	n/a
Other	n/a	One item concerning the fact that advertising letters are of interest*	n/a	n/a
**Facets that were not directly included in prior questionnaires**				
Interference with other life aspects	n/a	n/a	n/a	Q 6…that you have problems at work or school or in other areas due to your buying behavior?
				Q 13…that you have problems with other people due to your buying habits?
Resistance to PB	n/a	n/a	n/a	Q 12…that you try to limit your buying and can’t?
Degree of suffering	n/a	n/a	n/a	Q 5…that you suffer distress from your buying habits?

*Given that the GABS is copyrighted the original items were not listed.

^1^This item could also be assigned to the facet *withdraw*.

^2^This item could also be assigned to the facet *loss of control*.

The PBS varies from the GABS [28] in that way that the latter is basically based on the assumption that PB belongs to the behavioral addiction spectrum. Hence, the GABS includes many items concerning craving and preoccupation, whereas loss of control aspects are underrepresented. The use of the PBS could add value in future research projects considering both impulse control and addictive features of excessive buying behavior.

Contrary to the RCBS [24], several items of the new measure refer to negative consequences of PB given that they are part of the operationalized criteria for PB suggested by McElroy et al. [7] as well as the diagnostic criteria for other behavioral addictions [14]. The presence of negative consequences is a key characteristic of clinical relevant PB based on our experience with patients. Negative consequences should be considered as one facet (among others) associated to the maintenance of the pathology [8,9]. Becoming aware of the negative consequences may induce discomfort and negative feelings, which in turn can lead to buying binges, given the circumstance that buying is used to elevate negative mood states [9–11]. Consequently, we believe that a clinical screener for PB should include items assessing the negative outcomes of the pathological behavior.

Another advantage of the PBS is the inclusion of the supplementary MHE-items to obtain hints for meaningful additional assessments. With regard to the supplementary M-item which refers to symptoms of mania or hypomania, only 0.7% of our sample indicated having possible symptoms (answering “frequently” or “very frequently”). The low frequency seems plausible given a recently published interview-based representative study that reported the 12-months prevalence of bipolar disorder as 1% in Germany [44]. The number of those who answered the supplementary H-item with “frequently” or “very frequently” almost resembled the findings of an earlier study that examined compulsive hoarding in a German representative sample. In that prior study, the prevalence of compulsive hoarding was estimated to be 4.7% [45] compared to 4.3% of participants in the present sample who reported possible symptoms of hoarding.

All three supplementary items need further investigation. Nevertheless, ratings of these items as at least “frequently” may suggest the presence of hoarding disorder that often accompanies PB or may be helpful for the delineation from excessive buying within mania or buying with the primary goal of enrichment which would need subsequent, more fine-grained exploration. Future studies should consider the validation of the supplementary items by using external criteria such as a specific questionnaire for hoarding disorder, information on pre-diagnosed mania from patients’ charts or self-reported purchase data.

Last, the assessment of PB symptoms within a specific time frame and the consistent answering format (only frequencies) are strengths of the new measure. Taken together, we believe the PBS provides additional value beyond other PB measures to the assessment of PB, whereas the assumption that the PBS is superior to existing scales needs further empirical verification.

### Age and gender effects

With respect to the sociodemographic variables, we observed a negative correlation between age and PB as measured with both the PBS as well as the CBS. This inverse relationship is well known from previous studies that have used different PB questionnaires indicating a higher propensity for PB in younger age [2–6,24,27]. We also detected a weak gender effect for the PBS and its two subscales, but not for the CBS. Of note, possible gender differences in PB are still debated, given that population-based surveys revealed mixed results [2–6,26]. The PBS could contribute to further insights into the gender topic, especially when considering the factor *excessive buying behavior* that might be more sensitive to female participants as indicated by large effect sizes with respect to gender differences.

### Strengths and limitations

The use of a large-scale representative sample and its division into subsamples for subsequent analyses (i.e., EFA, CFA) are strengths of the study. Limitations of the present work include the lack of information on external criteria for PB (e.g., purchase records), divergent validity, retest reliability, and the comparison of the PBS with existing measures of PB other than the CBS. Moreover, the study did not include a group of individuals with clinically significant PB. These shortcomings should be addressed in further investigations.

## Conclusions

The PBS represents a new, useful measure for PB with good psychometric properties that may facilitate clinical screening and research on PB. Future studies involving treatment seeking patients with PB and other clinical samples are needed to investigate whether the two-factor structure can be replicated. The involvement of treatment samples would not only offer the opportunity to better define a valid cutoff point but also to explore its sensitivity and specificity as well as the sensitivity to change of the new scale.

## Supporting Information

S1 Table (DOCX)
