# Supplementary material for: The Pathological Buying Screener: Development and Psychometric Properties of a New Screening Instrument for the Assessment of Pathological Buying Symptoms
Source: PLoS One. 2015 Oct 21;10(10):e0141094. doi: 10.1371/journal.pone.0141094 (PMC4619303; doi:10.1371/journal.pone.0141094)
Supplement: S1 Table — (DOCX) [file pone.0141094.s001.docx]

**S1 Table. Factor loadings and means of the rated items for the initial 20-item Pathological Buying Screener, Study 1 (*n* = 498)**

| Item No. ^a^ | initial Item No. ^b^ | Item  English translation (German version) | Factor | | *Mean* | *SD* |
| --- | --- | --- | --- | --- | --- | --- |
|  |  |  | 1 | 2 |  |  |
|  |  | How often does it occur … (Wie oft kommt es vor, …) |  |  |  |  |
| Q6 | iQ11 | ...that you have problems at work or school or in other areas due to your buying behavior?  (dass Sie aufgrund Ihres Kaufverhaltens Probleme in beruflichen, schulischen oder anderen Lebensbereichen haben) | 0.973 | -0.169 | 1.14 | 0.51 |
| Q9 | iQ15 | ...that you hide your buying habits from others?  (dass Sie Ihr Kaufverhalten vor anderen verbergen) | 0.918 | -0.130 | 1.16 | 0.54 |
| Q13 | iQ20 | ...that you have problems with other people due to your buying habits?  (dass Sie mit anderen Menschen aufgrund Ihrer Kaufgewohnheiten Probleme haben) | 0.905 | -0.147 | 1.15 | 0.55 |
| Q12 | iQ19 | ...that you try to limit your buying and can’t?  (dass Sie versucht haben, ihr Kaufverhalten einzuschränken und es nicht schaffen) | 0.901 | -0.039 | 1.16 | 0.55 |
| Q11 | iQ18 | ...that you cannot stop buying things despite financial problems?  (dass Sie trotz finanzieller Probleme nicht aufhören können zu kaufen) | 0.888 | -0.063 | 1.16 | 0.53 |
| Q5 | iQ10 | ...that you suffer distress from your buying habits?  (dass Sie unter Ihren Kaufgewohnheiten leiden) | 0.866 | -0.060 | 1.16 | 0.53 |
| Q3 | iQ7 | ...that you have financial difficulties due to your buying habits?  (dass Sie durch Ihr Kaufverhalten unter finanziellen Problemen leiden) | 0.784 | 0.079 | 1.25 | 0.63 |
| Q2 | iQ5 | ...that you feel embarrassed when others ask you about your buying behavior?  (dass es Ihnen unangenehm ist, wenn andere Sie auf Ihr Kaufverhalten ansprechen) | 0.724 | 0.061 | 1.22 | 0.62 |
| Q8 | iQ14 | ...that at times you don’t feel good and that you feel better when you go buying?  (dass es Ihnen schlecht geht und sich das bessert, wenn Sie einkaufen) | 0.649 | 0.228 | 1.30 | 0.71 |
| Q1 | iQ8 | …that you can’t stop thinking about buying?  (dass Sie ständig ans Kaufen denken müssen) | 0.641 | 0.175 | 1.17 | 0.51 |
| ***--*** | iQ4 | …that you cannot avoid thoughts about buying?  (dass Sie Gedanken an das Einkaufen nicht unterdrücken können?) | 0.536 | 0.273 | 1.26 | 0.61 |
| -- | iQ6 | … that you feel a strong urge to buy something?  (dass Sie ein dringendes Verlangen verspüren, etwas zu kaufen?) | 0.531 | 0.372 | 1.39 | 0.76 |
| -- | iQ13 | … that you spend money which was actually meant for other purposes?  (dass Sie Geld fürs Einkaufen ausgeben, das eigentlich für andere Zwecke bestimmt war?) | 0.436 | 0.411 | 1.43 | 0.76 |
| -- | iQ3 | … that you spend your money instantly?  (dass Sie Ihr Geld sofort ausgeben?) | 0.415 | 0.412 | 1.45 | 0.79 |
| Q10 | iQ16 | ...that you buy more than you had planned?  (dass Sie mehr kaufen, als Sie sich vorgenommen haben) | -0.209 | 0.908 | 1.87 | 0.91 |
| Q7 | iQ12 | …that you buy more things than you need?  (dass Sie mehr Dinge kaufen als Sie benötigen) | -0.124 | 0.907 | 1.80 | 0.92 |
| Q4 | iQ9 | ...that you spend more time buying than you intended?  (dass Sie länger einkaufen als beabsichtigt) | -0.147 | 0.783 | 1.77 | 0.98 |
| -- | iQ2 | … that you have the irresistible desire to buy something?  (dass Sie den unwiderstehlichen Wunsch haben, etwas zu kaufen?) | 0.287 | 0.526 | 1.59 | 0.83 |
| -- | iQ17 | … that the things you have bought remain unused?  (dass die Dinge, die Sie gekauft haben, unbenutzt bleiben?) | 0.151 | 0.515 | 1.46 | 0.66 |
| -- | iQ1 | … that you buy to make yourself feel better?  (dass Sie einkaufen, um sich besser zu fühlen?) | 0.283 | 0.481 | 1.61 | 0.89 |

^a^ Item No. of the 13 Item final version of the PBS

^b^ initial Item No. of the 20 item version of the PBS before excluding items

Q = question number; iQ = initial question number

*Note.* The translation of the German version of items Q1 to Q13 into American English was performed by a licensed translator.
